# Throughput Improvement in Femtosecond Laser Ablation of Nickel by Double Pulses

**DOI:** 10.3390/ma14216355

**Published:** 2021-10-24

**Authors:** Kunpeng Chu, Baoshan Guo, Lan Jiang, Yanhong Hua, Shuai Gao, Jingang Jia, Ningwei Zhan

**Affiliations:** 1 Laser Micro/Nano Fabrication Laboratory, School of Mechanical Engineering, Beijing Institute of Technology, Beijing 100081, China; Kunpeng@bit.edu.cn (K.C.); jianglan@bit.edu.cn (L.J.); yanhong@bit.edu.cn (Y.H.); gaoshuai@bit.edu.cn (S.G.); jingang@bit.edu.cn (J.J.); ningwei@bit.edu.cn (N.Z.); 2 Yangtze Delta Region Academy of Beijing Institute of Technology, Jiaxing 314000, China; 3 Beijing Institute of Technology Chongqing Innovation Center, Chongqing 401120, China

**Keywords:** femtosecond laser, double pulses, nickel, two-temperature model

## Abstract

In this study, femtosecond laser double pulses were tested to improve their nickel ablation efficiency. The experimental results indicated that compared with single pulses, double pulses with different delay times generated craters with larger diameters and depths. The results obtained for three sets of double pulses with different energy ratios indicated that double pulses with an energy ratio of 1:9 had the highest ablation efficiency, followed by those with energy ratios of 2:8 and 5:5. The double pulses with the aforementioned three energy ratios achieved the maximum ablation efficiency when the delay time was 3–4 ps. Compared with single pulses, double pulses with an energy ratio of 1:9 generated craters with an up to 34% greater depth and up to 14% larger diameter. In addition, an interference effect was observed with a double pulse delay time of 0 ps, which has seldom been reported in the literature. The double pulses were simulated using the two-temperature model. The simulation results indicated that double pulses with an energy ratio of 1:9 with a delay time of 4 ps can perform the strongest ablation. These simulation results are in line with the experimental results.

## 1. Introduction

In recent decades, the development of pulse-shaping technology has provided greater opportunities for the application of ultrashort pulse laser technology. By integrating materials science, chemistry, physics, and other disciplines, femtosecond laser micro/nano processing technology has been extensively developed and applied in information-related fields, the chemical industry, aerospace, and the environmental industry [1,2,3,4,5,6,7]. Femtosecond lasers have an extremely high peak power (higher than 10^14^ W/cm^2^) and extremely short pulse width (approximately 10^−15^ s); thus, these lasers can process most materials. Materials processed using femtosecond lasers have an extremely small heat-affected zone (HAZ), which can safely be ignored [8,9]. Therefore, femtosecond lasers are increasingly used in micro/nano manufacturing.

Femtosecond lasers are widely used in metal processing due to their aforementioned advantages. For example, femtosecond lasers are used for producing high-quality and high-aspect-ratio micro-holes in metallic materials, especially those used in aircraft engines [10] and inertial-confinement nuclear fusion devices [11,12]. Nickel is a highly versatile metal, and femtosecond lasers have been used in the fabrication of various nickel structures [13,14,15]. In recent years, nickel has been widely used for fabricating the film-cooling holes of aerospace engine turbine blades. The air inlet passage in a turbine blade engine must contain film-cooling holes in order to enable the engine to operate stably. To ensure the normal operation and reliability of film-cooling holes in ultra-high temperature environments, advanced cooling technologies must be used, and these holes should be made of a nickel-based superalloy. Film-cooling holes have a small diameter (about 0.2–0.8 mm), high aspect ratio, and complex spatial angle; therefore, they must be processed with a high precision and efficiency. Many methods, such as the electric discharge machining method [16], coaxial water-jet-assisted machining method [17], electrochemical drilling method [18], and picosecond laser processing [19], have been used for processing the film-cooling holes of turbine blades; however, these methods are associated with problems such as a large heat-affected zone, low geometric accuracy, and uneven quality. Femtosecond lasers can solve these problems, but their processing efficiency is low. Determining a suitable method for improving the processing efficiency of femtosecond lasers when producing film-cooling holes is a critical problem. The main component of nickel-based superalloys is metallic nickel [10]. Zhang et al. [20] compared the depths and areas of the craters formed in nickel during femtosecond double-pulse (with the same energy ratio) and single-pulse irradiation. They found that the ablation efficiency of double pulses was higher than that of single pulse. Shen et al. [13] conducted an experimental study of collinear geometric double-pulse femtosecond LIBS’ on nickel samples in the ambient air in order to clarify the contribution process of the double-pulse signal enhancement observed compared with the single-pulse case. Donnelly et al. [21] performed a systematic study of the ultrafast ablation dynamics of nickel samples using the double-pulse configuration and discussed the changes in ablation efficiency with changes in the pulse delay time. Recently, there have also been studies [22,23] on promoting the ablation of metals using (near-)THz bursts of pulses, which usually include double-pulse splits. However, there have been few studies on the efficiency of the double-pulse ablation of nickel with femtosecond lasers with different energy ratios. Therefore, in-depth research must be conducted on this topic.

## 2. Materials and Methods

**Figure 1:**
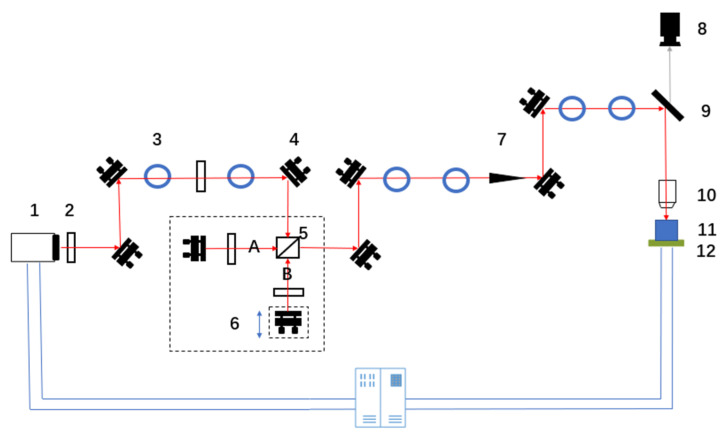
Schematic of the light path in the experiment (1—fs laser; 2—neutral density filter; 3—diaphragm; 4—mirror; 5—beam splitter; 6—one-dimensional linear translation stage; 7—shutter; 8—CCD camera; 9—dichroic mirror; 10—plano-convex lens; 11—sample; 12—six-degrees-of-freedom translation stage).

Figure 1 shows the light path in the experiment. A titanium sapphire femtosecond laser with an output wavelength of 800 nm, a pulse duration of 35 fs, a maximum output power of 5 W, and a maximum repetition frequency of 1 kHz was used in the experiment; the laser was provided by the Spectra Physics company based in the US. The size of the laser beam was adjusted using a diaphragm. The laser power was attenuated using a neutral density filter. In the light path, femtosecond laser beams were divided into two sub-pulses (A sub-pulse and B sub-pulse) by a beam splitter. Neutral-density filters were placed in the optical path of each sub-pulse. These filters were used to adjust the laser power of the two sub-pulses. The delay time of the two sub-pulses was adjusted by moving the one-dimensional linear translation stage back and forth. By adjusting a mirror, the two laser beams were converged to form a combined beam, and then focused on the sample, which was placed on a six-degrees-of-freedom translation stage. A computer was used to control the femtosecond laser’s processing by controlling the opening and closing of the shutter in the light path. By adjusting the rotation angle of a neutral density filter, the laser power could be adjusted. The six-degrees-of-freedom translation stage was produced by the Physik Instrumente (PI) company in Karlsruhe, Germany. This table had translation freedom along the X-direction, Y-direction, and Z-direction, and rotation freedom along the U-direction, V-direction, and W-direction. The minimum translation distance of the aforementioned table is 2 μm. A plano-convex lens (f = 100 mm) was used in the experiment. A charge-coupled device (CCD) camera was used for capturing images of the ablation of nickel by the femtosecond laser. The dimensions of the pure nickel samples used in the experiment were 20 × 20 × 1 mm^3^, and each nickel sample’s upper surface was mechanically polished such that their upper surface roughness was 10 nm. Finally, the nickel sample was in an air atmosphere during the experiment. The optical microscope (OM) was provided by the Olympus company, and the atomic force microscope (AFM) was provided by the Bruker company.

The steps for finding the zero delay point in the double-pulse configuration are as follows. First, to determine the spatial coincidence of the double pulses, with only the B sub-pulse blocked, by outlining the image formed by the CCD camera of the crater ablated by the A sub-pulse on the surface of the nickel with a circle. Then, block only the A sub-pulse, outline the crater ablated by the B sub-pulse with a circle, and finally rotate the knob of the mirror behind the B sub-pulse so that the crater mark formed by the B sub-pulse coincides with the A sub-pulse. This completes the double pulses’ spatial coincidence. The next task is to produce the time coincidence of the double-pulse configuration: leave the position of the A sub-pulse mirror unchanged, move the translation stage to change the position of the mirror behind the B sub-pulse, and place a piece of fluorescent paper in the position at which the two sub-pulses’ beams are combined. Adjust the one-dimensional translation stage until the most obvious light and dark stripe structure appears on the fluorescent paper, which can be judged to be the time coincidence. After completing the last two steps, confirm that the zero delay of the double pulses has been set.

**Figure 2:**
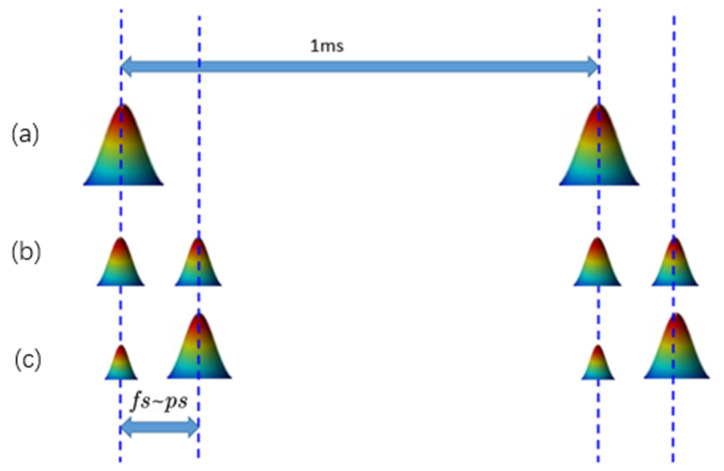
Schematic of the pulses used for ablation, with a single pulse and with double pulses with varying energy ratios: (**a**)—single pulse; (**b**)—a double pulse with an energy ratio of 5:5; (**c**)—double pulses with a different energy ratio (1:9 or 2:8). The maximum repetition rate of the laser was 1 kHz.

The femtosecond laser single-pulse ablation of the nickel was completed by blocking one of the sub-pulses. The double-pulse experiment was conducted under various delay times and energy ratios. When the energy of the two sub-pulses was different, the double-pulse processing mode in which a low- or high-power laser pulse is in front is called the low–high or high–low double-pulse mode, respectively. One study [24] proved that the ablation efficiency of the low–high double pulse-mode is higher than that of the high–low double-pulse mode; therefore, the low–high double-pulse mode was selected in the experiment. In order to make the differences between the craters’ results with different ratios of double pulse more obvious, three energy ratios of double pulse were selected for the experiments (1:9, 2:8, and 5:5). The sum of the energy of the double pulses of the three laser fluence ratios remained the same as that of the single pulse. The three double-pulse processing modes and the single pulse processing mode are illustrated in Figure 2.

## 3. Results

**Figure 3:**
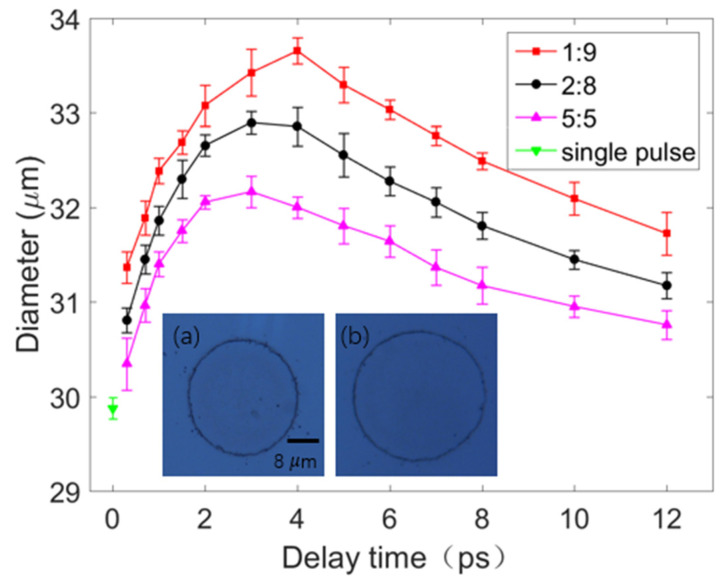
A comparison of the diameters of the craters ablated by the double pulses (with three energy ratios) and single pulse. The red, black, and pink lines represent the results obtained for double pulses with energy ratios of 1:9, 2:8, and 5:5, respectively. The downward green triangle represents the results obtained for the single pulse. Inset (**a**)—image of a crater ablated by the single pulse; inset (**b**)—image of a crater ablated by double pulses with an energy ratio of 1:9 and a delay time of 4 ps. Insets (**a**,**b**) have the same scale bar.

**Figure 4:**
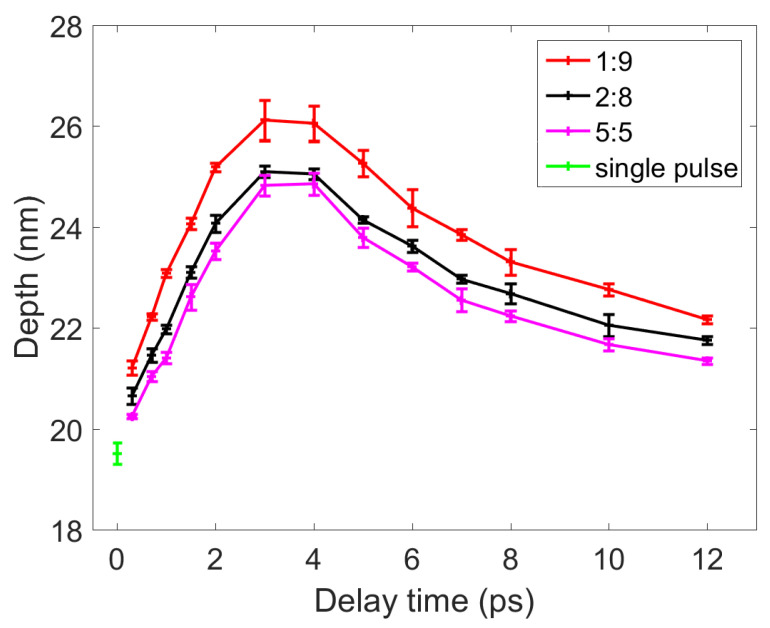
A comparison of the depths of the craters ablated by the double pulses (with three energy ratios) and single pulse. The red, black, and pink lines represent the results obtained for double pulses with energy ratios of 1:9, 2:8, and 5:5, respectively. The downward green triangle represents the results obtained for the single pulse.

Single-shot ablation experiments were conducted on the surface of nickel by a femtosecond single pulse (F = 1.0 J/cm^2^) and double pulses (F_1_ + F_2_ = 1.0 J/cm^2^) with three energy ratios. The diameter and depth of the ablated craters were characterized using the OM and AFM. When measuring the diameter of the craters, the magnification of the OM was 100×. When measuring the depth of the craters, the area scanned by the AFM was 50 × 50 μm^2^. Each group of experiments with different parameters (delay time or laser fluence ratio) was repeated 6 times, and the diameters and depths were measured using OM and AFM, respectively, and rounded to obtain the average value. The results of the OM and AFM are displayed in Figure 3 and Figure 4, respectively. In addition, when the sum of the double-pulse or single-pulse laser fluence was 0.5 J/cm^2^, the circle (as shown in Figure 3a) was not seen in the nickel sample.

The results displayed in Figure 3 indicate that the maximum diameters of the craters ablated by double pulses with energy ratios of 1:9, 2:8, and 5:5 were 33.66, 32.90, and 32.17 μm, respectively. These maximum diameters were obtained when the delay time was 3–4 ps. The diameter of the craters ablated by the single pulse was 29.65 μm. Thus, the diameter of the craters ablated by the double pulse with a delay time of 4 ps and an energy ratio of 1:9 was up to 14% higher than that of craters ablated by the single pulse. Similarly, as displayed in Figure 4, the maximum depths of the craters ablated by double pulses with energy ratios of 1:9, 2:8, and 5:5 were 26.12, 25.10, and 24.86 nm, respectively. These maximum depths were obtained when the delay time of the double pulses was 3–4 ps. The depth of the crater ablated by the single pulse was 19.51 nm; thus, the depths of the craters ablated by double pulses with a delay time of 3 ps and an energy ratio of 1:9 was up to 34% higher than that of the crater ablated by the single pulse.

**Figure 5:**
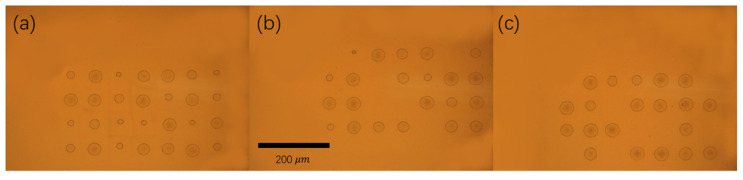
The results of the ablation experiments when the double-pulse delay time was 0 ps: (**a**)—energy ratio of 1:9; (**b**)—energy ratio of 2:8; (**c**)—energy ratio of 5:5. Panels (**a**–**c**) have the same scale. The scale bar is 200 μm.

**Figure 6:**
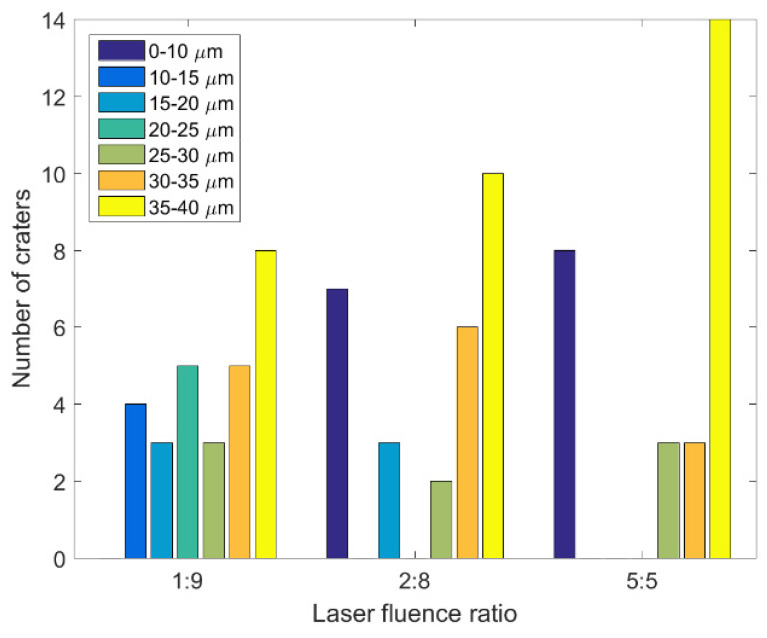
Diameter range histograms of the craters ablated by double pulses with three laser fluence ratios when the delay time was 0 ps.

Typically, when the laser fluences of single and double pulses are the same (F = F_1_ + F_2_), the ablation result obtained with a double-pulse delay time of 0 ps is close to that obtained for a single pulse. However, some unusual phenomena were observed in this study’s double-pulse ablation experiments. As displayed in Figure 5, 28 craters were ablated when the delay time of the double pulses with three energy ratios was 0 ps. The diameters of these craters were measured using the OM, and the corresponding results are presented in Figure 6 and Table 1. The diameters of the craters ablated by double pulses with an energy ratio of 1:9 were concentrated in the middle area (10–35 μm). At the aforementioned energy ratio, craters were formed in all 28 experiments, and eight of the craters had a diameter of more than 35 μm. When the energy ratio of the double pulses was 5:5, 20 craters were formed over the 28 experiments. At total of 14 of these craters had a diameter of more than 35 μm. Finally, when the energy ratio of the double pulses was 2:8, 22 craters were formed over the 28 experiments. At total of 10 of these craters had diameters exceeding 35 μm. The formation of the craters was influenced by the interference of double pulses when the delay time was 0 ps. The double pulses’ interference was strongest when their energy ratio was 5:5; therefore, the crater diameter had the widest distribution with the lowest proportion of values in the middle range (10–35 μm) when the energy ratio of the double pulses was 5:5. It was proved that the processing effect of double pulses at zero delay is obviously different from that of a single pulse, and this result can be used to develop multi-scale interference processing. In order to avoid the interference phenomenon of double pulses, vertical polarization optical pulse processing, achieved by changing the polarization of one of the double pulses, can also be used.

## 4. Discussion

The two-temperature model (TTM) was used to explain the different ablation efficiencies obtained in the experiment for the single pulses and double pulses with varying energy ratios.

The TTM describes the heat transfer between the laser heat source, material electrons, and material lattice during the laser ablation process using the following equations:

(1) $$C_{e}\left[T_{e},T_{l}\right]\frac{\partial T_{e}}{\partial t}=\nabla \left(K_{e}\left[T_{e},T_{l}\right]\nabla T_{e}\right)-G\left[T_{e},T_{l}\right]\left(T_{e}-T_{l}\right)+Q$$

(2) $$C_{l}\left[T_{e},T_{l}\right]\frac{\partial T_{l}}{\partial t}=G\left[T_{e},T_{l}\right]\left(T_{e}-T_{l}\right)$$

where the subscripts e and l represent the electron and lattice parameters, respectively; $C_{e}$ and $C_{l}$ represent the heat capacities of the electrons and lattices, respectively, and are regarded as constants; G is the electron–lattice coupling coefficient; Q is the average fluence of the incident laser light; and $K_{e}$ is the electronic thermal conductivity, which is expressed as follows [25]:

(3) $$K_{e}=K_{0}\times \frac{T_{e}}{T_{l}}$$

In the aforementioned equation, $K_{0}$ is the heat transfer coefficient of the electronic thermal conductivity. The parameter Q is expressed as follows [26]:

(4) $$Q\left(x,t\right)=\sqrt{\frac{4ln2}{\pi }}\frac{1-R\left(t\right)}{\delta \left(t\right)+\delta_{b}}\overset{n}{\underset{i=1}{\sum }}\frac{F_{i}}{t_{pi}}\{\left(\frac{x}{\delta \left(t\right)+\delta_{b}}\right)-4ln2{\left[\frac{t-2t_{pi}-\left(i-1\right)\Delta }{t_{pi}}\right]}^{2}\}$$

where $\delta_{b}$ is the depth of the ballistic transportation, δ(t) is the temperature-dependent depth of the ballistic transportation, R(t) is the temperature-dependent reflectivity, $F_{i}$ is the laser energy density of the incident laser beam, $t_{pi}$ is the pulse width of the incident laser beam (full width at half maximum), x is the depth from the sample surface, and Δ is the delay time of the subsequent laser pulse.

The parameters R(t) and δ(t), respectively, are expressed as follows [26]:

(5) $$R\left(T_{e},T_{l},\omega \right)=\frac{[R_{e}\left(n_{c}\right)-1{]}^{2}+[I_{m}\left(n_{c}\right){]}^{2}}{[R_{e}\left(n_{c}\right)+1{]}^{2}+[I_{m}\left(n_{c}\right){]}^{2}}$$

(6) $$\delta \left(T_{e},T_{l},\omega \right)=\frac{c}{2\cdot \omega \cdot I_{m}\left(n_{c}\right)}$$

where $R_{e}$($n_{c}$) represents the real part of the complex refractive index $n_{c}$, $I_{m}\left(n_{c}\right)$ represents the imaginary part of the complex refractive index $n_{c}$, c represents the speed of light in a vacuum, and ω represents the frequency of the femtosecond laser beam.

The relationship between the complex refractive index $n_{c}$ and the temperature-dependent complex dielectric function ε is expressed as follows [27]:

(7) $$n_{c}=\sqrt{\varepsilon }=\sqrt{\varepsilon_{1}+i\varepsilon_{2}}$$

where $\varepsilon_{1}$ and $\varepsilon_{2}$ are the real and imaginary parts of the complex dielectric function ε, respectively. The temperature-dependent complex dielectric function ε is expressed as follows [28]:

(8) $$\varepsilon =1-\frac{\omega_{p}^{2}}{\omega^{2}+\nu_{m}^{2}}+i\frac{\nu_{m}}{\omega }*\frac{\omega_{p}^{2}}{\omega^{2}+\nu_{m}^{2}}$$

where $\omega_{p}$ is the plasma frequency of the material and $\nu_{m}$ is the total electron scattering rate. The aforementioned parameters are expressed using Equations (9) and (12), respectively [29]:

(9) $$\omega_{p}=\sqrt{\frac{e^{2}\cdot n_{e}}{\varepsilon_{0}\cdot m_{e}}}$$

(10) $$\tau_{e-e}=\frac{1}{A_{e}\cdot T_{e}^{2}}$$

(11) $$\tau_{e-l}=\frac{1}{B_{l}\cdot T_{l}}$$

(12) $$\nu_{m}=\frac{1}{\tau_{e-e}}+\frac{1}{\tau_{e-l}}$$

The parameter $\tau_{e-e}$ is the electron–electron relaxation time; $\tau_{e-l}$ is the electron–lattice relaxation time; $A_{e}$ and $B_{l}$ are material constants for the electron relaxation time; $\varepsilon_{0}$ is the vacuum dielectric constant; and $n_{e}$, $m_{e}$, and e are the electron density, electron mass, and electron charge, respectively. The parameter electron–lattice coupling coefficient G is expressed using Equation (13) [30], where $G_{0}$ is the electron–lattice coupling strength at room temperature.

(13) $$G=G_{0}\left[\frac{A_{e}}{B_{l}}\left(T_{e}-T_{l}\right)+1\right]$$

The electron density is expressed as follows:

(14) $$n_{e}=\frac{N\cdot N_{A}\cdot \rho }{Y}$$

where N is the number of valence electrons of the material, $N_{A}$ is the Avogadro constant, ρ is the material density, and Y is the atomic weight of the material.

The finite-element method was used in this study to solve Equations (1) and (2). The initial temperatures of the nickel material’s electrons and lattice were assumed to be equal to the ambient temperature—namely, 300 K. The physical parameter values of nickel are listed in Table 2.

**Figure 7:**
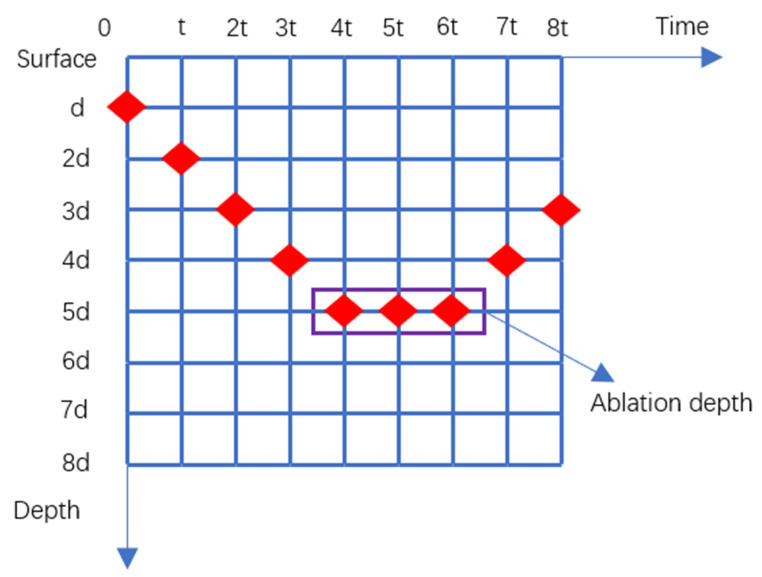
The process of calculating the depth of nickel ablation.

Some studies have indicated that materials can be ablated only when their lattice temperatures reach the following three temperatures: their melting point [35], vaporization temperature [36], or thermodynamic critical temperature [37]. In our simulation, when the lattice temperature exceeds the melting point of a material, the material is ablated. Figure 7 shows the process of calculating the depth of a nickel sample ablated by a femtosecond laser. The minimum unit of the time grid divided by the TTM is t and the minimum unit of the depth grid is divided by d. The ablation depth of the nickel sample is calculated at t, 2t, 3t, etc. When there is a maximum value and an inflection point in the depth value of the ablated nickel sample, the ablation depth of the sample under this parameter (specific delay time and fluence ratio) can be determined. After calculating the ablation depth and the maximum temperature of the lattice under each set of parameters, it was found that the ablation depth is proportional to the maximum lattice temperature. The ablation efficiency of a material is related to the maximum temperature of its lattice [22]. Therefore, only the maximum lattice temperature of the nickel surface must be known in order to determine the ablation efficiency.

**Figure 8:**
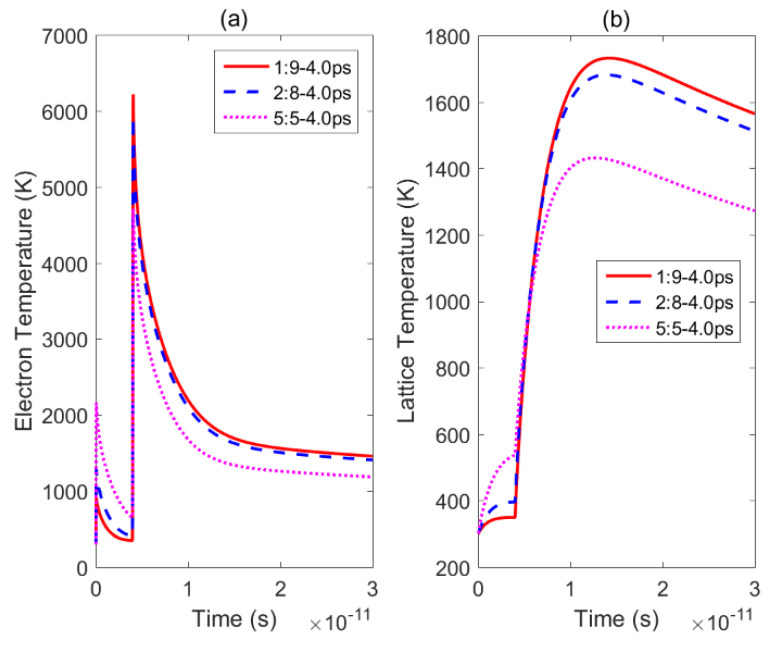
Changes in the lattice and electron temperatures over time under irradiation by double pulses with three energy ratios: (**a**)—electron temperatures obtained by irradiation with double pulses with a delay time of 4.0 ps and energy ratios of 1:9 (solid red line), 2:8 (dotted blue line), and 5:5 (dotted pink line); (**b**)—lattice temperatures obtained by irradiation with double pulses with a delay time of 4.0 ps and energy ratios of 1:9 (solid red line), 2:8 (dotted blue line), and 5:5 (dotted pink line).

**Figure 9:**
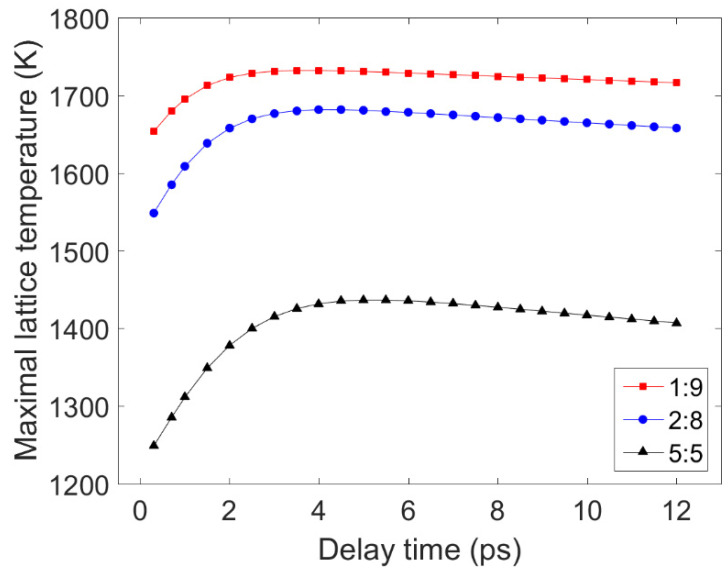
Changes in the maximum lattice temperatures with respect to the delay time for irradiation by double pulses with three energy ratios. The solid red, blue, and black lines represent the results obtained using double pulses with energy ratios of 1:9, 2:8, and 5:5, respectively.

Figure 8 displays the electron and lattice temperature trends simulated using the TTM using MATLAB under irradiation with three sets of double pulses. The position in the sample, where the temperature evolution was calculated, is the irradiated front surface of the nickel. Figure 9 shows the maximum lattice temperatures obtained using double-pulse irradiation with varying delay times and energy ratios. For the three energy ratios considered, the lattice temperature reached its maximum value when the delay time was 4.0 ps, 4.0 ps, and 5.0 ps, respectively. In Figure 9, it can be seen that the maximum temperature of the lattice formed by the double pulses with a laser fluence ratio of 1:9 was greater than the maximum temperatures reached using the other two ratios with the same time delay. So, the ablation efficiency was greatest when the energy ratio was 1:9 and the smallest when the energy ratio was 5:5. The ablation efficiency of a double pulse with an energy ratio of 5:5 was stronger than that of a single pulse, which has been confirmed in the literature [20]. Therefore, this result is consistent with the experiment results presented in Section 3.

## 5. Conclusions

In this study, single-shot ablation experiments were conducted on nickel samples using a femtosecond laser. The experimental results indicated that the double pulses with an energy ratio of 1:9 had the highest ablation efficiency, followed by those with energy ratios of 2:8 and 5:5. When the laser energy was the same, all the ablation efficiencies attained using double pulses were higher than those attained using a single pulse. The experimental results also indicated that the ablation efficiency (depth and diameter of the craters) of the double pulses with the aforementioned three energy ratios was the highest when the delay time was 3–4 ps. Moreover, when the delay time was 0 ps, the double pulses’ interference was the strongest at an energy ratio of 5:5. Finally, by changing the delay time of the double pulses, the maximum temperature of a sample’s lattice surface could be adjusted and different ablation depths could be obtained, which verified the experimental results. This research provides theoretical and experimental guidance for the efficient micro/nano processing of nickel-based materials, such as producing the film-cooling holes of turbine blades.

## Figures and Tables

**Table 1:** The distribution of the ablation craters’ diameters under a double-pulse delay time of 0 ps.

Diameter	1:9	2:8	5:5
0~10	0	25.0%	28.6%
10~15	14.2%	0	0
15~20	10.7%	10.7%	0
20~25	17.9%	0	0
25~30	10.7%	7.2%	10.7%
30~35	17.9%	21.4%	10.7%
35~40	28.6%	35.7%	50.0%

**Table 2:** The values of various parameters in the TTM.

Symbol	Value
$C_{e}$	$1065\;J\cdot m^{-3}\cdot K^{-1}$ [31]
$C_{l}$	$4.1\;\times \;10^{6}\;J\cdot m^{-3}\cdot K^{-1}$ [30]
$G_{0}$	$3.6\;W\cdot m^{-1}\cdot K^{-1}$ [32]
$K_{0}$	$90\;W\cdot m^{-1}\cdot K^{-1}$ [31]
$\delta_{b}$	13.5 nm [33]
$t_{pi}$	35 fs
c	$3\;\times \;10^{8}m\cdot s^{-1}$
ω	c /800 nm
e	$-1.6\;\times \;10{}^{-19}\;C$
$\varepsilon_{0}$	$8.85418717\;\times \;10{}^{-12}\;F\cdot m^{-1}$
$m_{e}$	$9.1\;\times \;10{}^{-31}\;\mathrm{Kg}$
N	2
$N_{A}$	6.022 × 10^−23^
ρ	$8.88\;{g*\mathrm{cm}}^{-3}$
Y	59
$A_{e}$	$0.59\;\times \;10{}^{-7}\;s^{-1}\cdot K^{-2}$ [31,34]
$B_{l}$	$1.4\;\times \;10^{11}s^{-1}\cdot K^{-1}$ [31,34]

## Data Availability

The data presented in this study are available on request from the corresponding author.
